# The lncRNA Punisher Regulates Apoptosis and Mitochondrial Homeostasis of Vascular Smooth Muscle Cells via Targeting miR-664a-5p and OPA1

**DOI:** 10.1155/2022/5477024

**Published:** 2022-05-25

**Authors:** Yanyan Yang, Min Li, Yan Liu, Zhibin Wang, Xiuxiu Fu, Xingqiang He, Qi Wang, Xiao-xin Li, Huibo Ma, Kun Wang, Lu Zou, Jian-xun Wang, Tao Yu

**Affiliations:** ^1^Department of Immunology, School of Basic Medicine, Qingdao University, Qingdao 266071, China; ^2^Institute for Translational Medicine, The Affiliated Hospital of Qingdao University, 266021, China; ^3^Department of Cardiac Ultrasound, The Affiliated Hospital of Qingdao University, 266000, China; ^4^Department of Cardiology, The Affiliated Hospital of Qingdao University, 266000, China; ^5^Department of Vascular Surgery, The Affiliated Hospital of Qingdao University, Qingdao, China; ^6^Department of Cardiovascular Surgery, The Affiliated Hospital of Qingdao University, Qingdao, China

## Abstract

Long noncoding RNAs (lncRNAs) are important regulators of various cellular functions. Recent studies have shown that a novel lncRNA termed Punisher is highly expressed in cardiovascular progenitors and has potential role in cardiovascular diseases. However, its role, especially in molecular mechanism, is unclear. In our present study, we observed that Punisher was obviously downregulated in atherosclerotic plaques. Further research proved that it can suppress the apoptosis of VSMCs potentially contributing to the progression of atherosclerosis. Intriguingly, Punisher revealed to regulate mitochondria fission as well as mitochondrial functions induced by hydrogen peroxide (H_2_O_2_) in VSMCs. Mechanistically, Punisher was further proved to serve as a ceRNA which directly binds to miR-664a-5p and consequently regulates its target OPA1, and finally contributes to the biological function of VSMCs. Particularly, Punisher overexpression distinctly suppressed neointima formation and VSMC apoptosis in vivo. Encouragingly, these results were in accordance with findings obtained with the clinical evaluation of patients with atherosclerosis. Our data provides the significant relationship among OPA1, mitochondrial homeostasis, VSMC apoptosis, and atherosclerosis. And lncRNA Punisher and miR-664a-5p could serve as the novel and potential targets in the diagnosis and treatment of cardiovascular diseases.

## 1. Introduction

Atherosclerosis (AS) is a systemic, diffuse, and progressive chronic vascular disease, characterized by lipid deposition in some parts of the arteries, abnormal proliferation, and migration of vascular smooth muscle cells (VSMCs) and fibrous matrix and excessive apoptosis of VSMCs. These abnormalities result in the development of the atherosclerotic plaque, a fibrous plaque that gradually dilates into the blood vessels, inducing vascular stenosis [1–4].

Apoptosis happens at various stages of AS and is the main factor leading to the instability of the atherosclerotic plaques. VSMCs are one of the main components of the vascular wall, and also, the apoptosis of these cells is involved in the development of the atherosclerotic plaques. Emerging evidences suggested that the excessive proliferation and apoptosis of VSMCs plays the critical role in the occurrence, development, and instability of AS [5]. In the physiological state, VSMC renewal happens at a relatively slow speed; in the late-stages of atherosclerosis, instead, VSMC apoptosis is prominent, contributing to the instability of the plaque and increasing the probability of thrombosis. Additionally, several studies indicate that VSMC apoptosis not only accelerates atherosclerosis but also promotes calcification and medial degeneration of plaque, prevents expansion of remodeling, and promotes the formation of atherosclerotic stenosis [5]. Conversely, in the early stage, the abnormal VSMCs exhibited excessive proliferation. Therefore, the imbalance between VSMC proliferation and apoptosis is considered to be the important factor determining the progression of atherosclerosis. The regulation of the apoptosis in VSMC can prevent the increase in size and calcification of late atherosclerotic plaques and might serve as a potential therapeutic target. Intriguingly, increase evidence reported that mitochondrial dynamics is crucially involved in intrinsic pathway of apoptosis which results in regulation of energy supply, cellular respiration, signal transduction, and cell cycle [6, 7]. Therefore, the study of the mechanisms of mitochondrial division and fusion in the process of VSMC apoptosis might allow the discovery of new drug targets effective in treatment of AS.

To date, constitutive exploration suggested that noncoding RNAs are proved to be significant regulators of various cellular processes, in particular, cell apoptosis, and mitochondrial dynamics [8–18]. Long noncoding RNAs (lncRNAs) are longer than 200 nucleotides, do not encode proteins, and might be located in the nucleus or cytoplasm. Most lncRNAs have conserved secondary structures, specific splicing patterns, and subcellular localization. The specificity of lncRNAs indicates that they are functional. However, the regulatory role of lncRNAs is difficult to determine because it is not only based on their sequence, structure, and conservation. Although research on lncRNAs has rapidly progressed in recent years, the function and molecular mechanism of most lncRNAs remain unclear. Several studies have shown that lncRNAs play an important role not only in cell proliferation, differentiation, and aging, but also in pathological processes, such as cancer, cardiovascular disease, neurodegenerative disease, and autoimmune diseases [17, 19–23]. Moreover, several important studies have indicated that lncRNA may be closely related to the function of heart and blood vessels [24–29].

Punisher is previously identified as a conserved lncRNA in animal and humans and detected previously in mature endothelial cells [30, 31]. Punisher appears to be potentially involved in the regulation of cell-cycle, vascular development, DNA damage, and chromatin modification. Punisher also acts as a suppressor of branching and vessel formation. All these evidences indicated that Punisher has potential role in cardiovascular diseases. However, the biological function and molecular mechanism of Punisher in AS remain unclear.

In this study, we investigated the role of Punisher in the pathogenesis of AS, in relation to the VSMC apoptosis. We demonstrated that Punisher was more expressed in VSMCs than in other cells which indicate it could play more significant role in VSMCs. Through bioinformatics analysis, we found that Punisher could serve as a competitive endogenous RNA (ceRNA) to regulate the downstream miR-664a-5p. Furthermore, we explored the role of the mitochondrial fusion protein optic atrophy (OPA)1 in the regulation of VSMC's biological function. In vivo test demonstrated the significant role of Punisher in the treatment of atherosclerosis. These findings improve the understanding of the mechanisms that regulate VSMC apoptosis and mitochondrial dynamics and provide novel insight into the pathogenesis of atherosclerosis.

## 2. Results

### 2.1. Punisher Regulates Apoptosis and Mitochondrial Fission Induced by H_2_O_2_ in VSMCs

**Figure 1:**
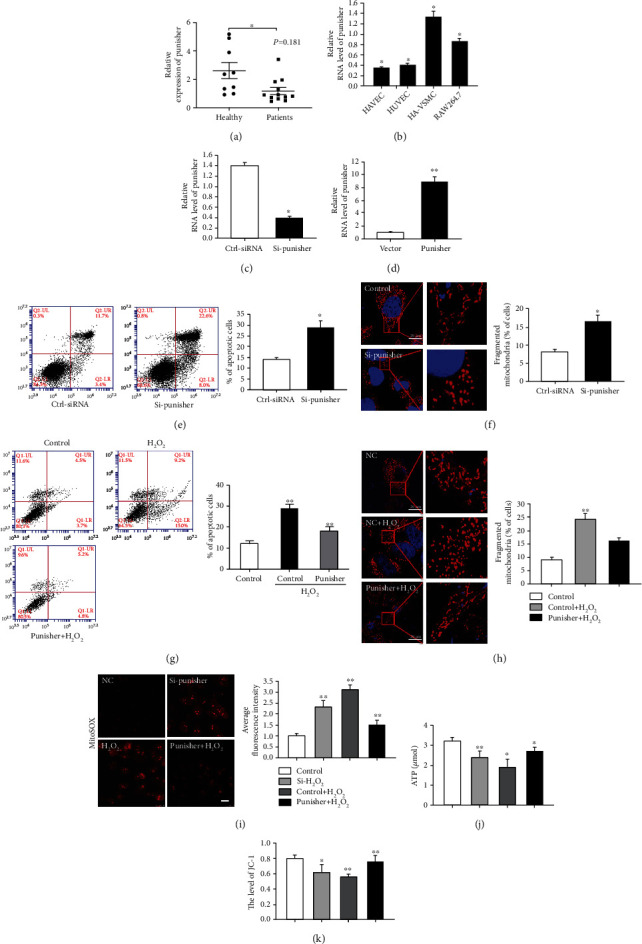
Punisher regulates cell apoptosis and mitochondria dynamics of VSMC. (a) Punisher transcript expression in atherosclerotic plaques of patients (*n* = 12) and healthy people (*n* = 9) was measured by quantitative reverse transcription–polymerase chain reaction (qRT-PCR). (b) qRT-PCR analysis of Punisher mRNA expression in human arterial vascular smooth muscle cells (HA-VSMC), RAW264.7, and endothelial cells. (c, d) siRNAs and overexpression plasmids were designed and transfected for 24 hours to knock down or enforce Punisher expression in VSMCs. The expression of Punisher was quantified by qRT-PCR. (e) Annexin V-FITC/propidium iodide staining and FACS quantification of the number of apoptotic cells in HA-VSMC after transfection of si-Punisher. (f) MitoTracker staining of mitochondrial fission in VSMC after Punisher knockdown. (g) Annexin V-FITC/propidium iodide staining and FACS quantification of the number of apoptotic cells in HA-VSMC after transfection of Punisher overexpression plasmids with H_2_O_2_ stimulation. (h) MitoTracker staining of mitochondrial fission in VSMCs after Punisher overexpression with H_2_O_2_ stimulation. (i, j) Evaluation of ROS and ATP production after knockdown or overexpression of Punisher with or without H_2_O_2_ treatment. (k) Measurement of mitochondrial membrane potential by detecting mitochondrial membrane potential expression using JC-1. All values are the average of at least 3 biological replicates, and data shown are the mean ± SD. Scale bars: 20 *μ*m. ^∗^*p* < 0.05; ^∗∗^*p* < 0.01.

To evaluate the role of Punisher in AS, we first detected the expression of Punisher in the atherosclerotic aortas of patients. Interestingly, we observed that Punisher expression was obviously lower in the atherosclerotic arteries compared with those of healthy people (Figure 1(a) and Figure S1), indicating that Punisher may be a potential regulator in the development of AS. Next, we examined the expression of Punisher in various cells related to AS and observed that VSMC had the highest Punisher levels compared with the other cell types (Figure 1(b)). Therefore, we further explored the functional role of Punisher using the human artery VSMC line. We used siRNA and overexpression plasmids to suppress or increase Punisher expression and verified by qRT-PCR (Figures 1(c) and 1(d)). Then, we found that Punisher can slightly regulate the proliferation (Figure S2a) and migration of VSMC cells (Figure S2b-c). Therefore, we further consider whether there are simultaneous other functions regulated by Punisher.

Previous studies have demonstrated that apoptosis is an independent risk factor for atherosclerotic lesions, and excessive apoptosis in the atherosclerotic plaques plays an important role in the formation of unstable plaques [32]. Meanwhile, aberrant mitochondrial fusion and fission are crucially involved in the regulation of apoptosis [33]. Therefore, we explored whether Punisher participates in the mediation of VSMC apoptosis and mitochondrial dynamics. Additionally, hydrogen peroxide (H_2_O_2_) induces both apoptosis and mitochondrial fission in VSMCs and has been used to generate oxidative stress mimicking atherosclerosis [34–36]. Previous study has shown that the response to oxidative stress induced by H_2_O_2_ in VSMC depends upon the concentration of H_2_O_2_ applied (Figure S3) [37]. We first investigated whether downregulation of Punisher is involved in the occurrence of apoptosis and mitochondrial fission. We found that si-Punisher transfection strongly promoted VSMC apoptosis and mitochondrial fission (Figures 1(e) and 1(f)), whereas upregulation of Punisher displayed the opposite effects upon H_2_O_2_ treatment (Figures 1(g) and 1(h)). Consistently, further studies revealed that the knockdown of Punisher promoted ROS production (Figure 1(i)). The production of ATP and mitochondrial membrane potential were also positively regulated by Punisher (Figures 1(j) and 1(k)). Besides, since increased sensitivity to H_2_O_2_ could also be explained by a reduced activity of antioxidants, we detected whether Punisher can regulate antioxidant enzymes and found that it could practically activate the expression of SODs and GPx (Figure S4). Taken together, these data indicated that Punisher can partially induce cell proliferation and migration, and most importantly, it suppresses apoptosis and mitochondrial dynamics in VSMCs which might finally result in the protection of atherosclerosis.

### 2.2. Punisher Regulates the Expression of OPA1

**Figure 2:**
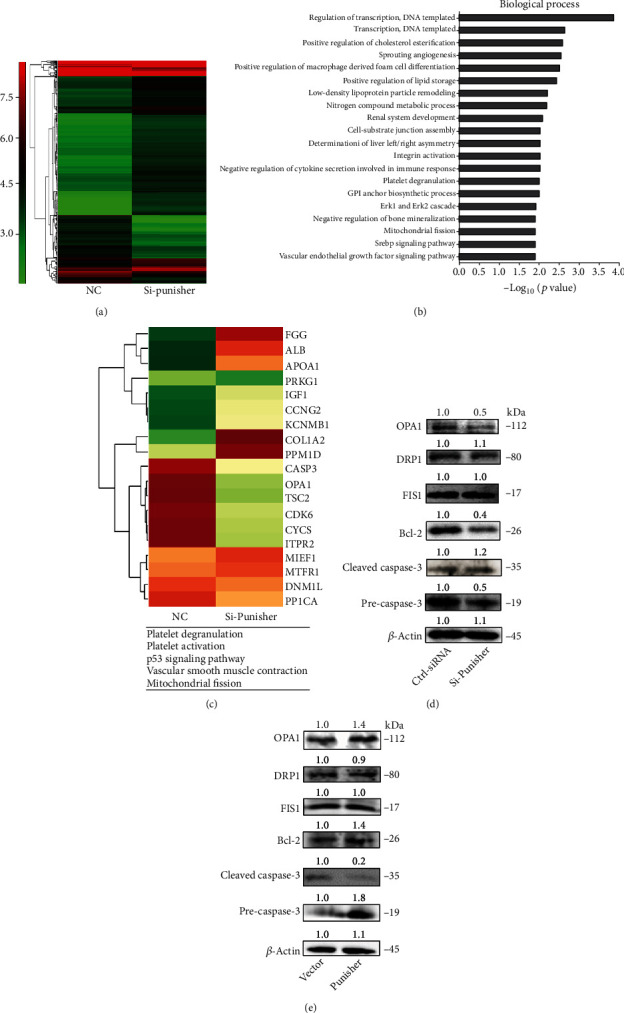
Punisher is involved in the signaling pathway of cell apoptosis and mitochondrial fission. (a) Hierarchical clustering analyses of 331 upregulated and downregulated 274 genes in si-Punisher samples relative to control-siRNA (Cntl-siRNA). (b) GO analysis in biological process after si-Punisher transfection for 24 hours in VSMCs. (c) Heat map of main downstream genes that are regulated in Punisher knockdown samples. (d, e) Mitochondrial proteins and apoptosis proteins were determined by western blotting. All values are the average of at least 3 biological replicates, and data shown are mean ± SD.

To understand the molecular mechanism through which Punisher regulates cell apoptosis and mitochondrial fission, we executed unbiased microarray analysis to survey gene expression alteration regulated by Punisher in VSMCs. We found that 331 and 274 genes were upregulated and downregulated more than 2 folds (*p* < 0.05), respectively, when Punisher was knocked down (Figure 2(a)). Gene ontology analysis indicated that the downregulated genes were mostly related to cell apoptosis, mitochondrial fission, platelet degranulation, platelet activation vascular smooth muscle contraction, and p53 signaling pathway (Figure 2(b)). Further analysis showed that many of the downregulated genes, such as caspase-3 and OPA1, were involved in apoptosis and mitochondrial signaling (Figure 2(c)). Then, we investigated the mitochondrial genes by using western blot and found that knockdown of Punisher could significantly decrease OPA1 expression whereas there was no or slight effect of other mitochondrial fission and fusion proteins, indicating that OPA1 could be the potential downstream target of Punisher (Figure 2(d)). Meanwhile, Bcl-2 and cleaved caspase-3 were also suppressed after knocking down of Punisher and activated by overexpression of Punisher (Figure 2(e)).

### 2.3. Punisher Regulates miR-664a-5p Expression and Activity

**Figure 3:**
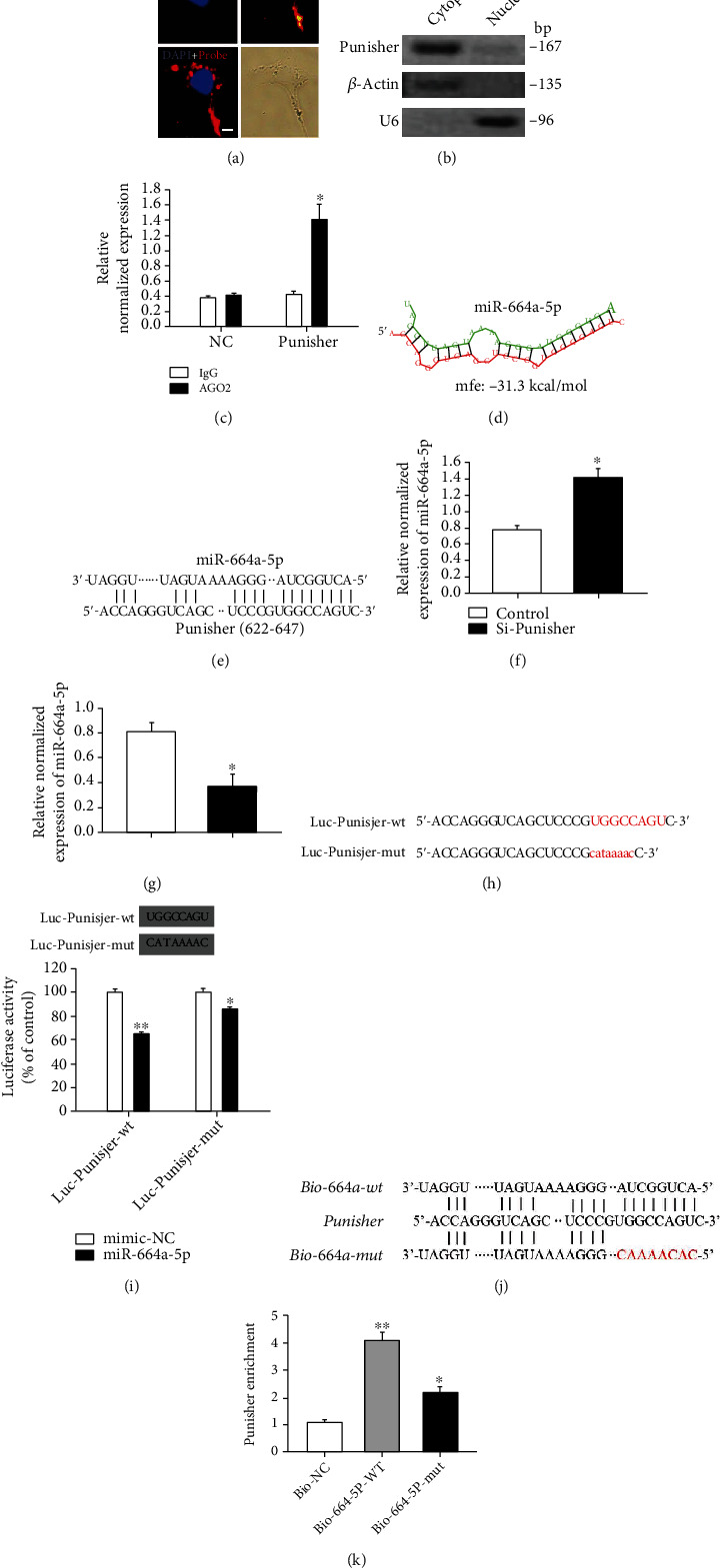
Punisher serves as a miRNA sponge for miR-664a-5p. (a, b) Cellular localization of Punisher in VSMCs by using FISH and western blotting. (c) Assessment of binding potential between Punisher and AGO2 through RIP assay. (d, e) Schematic of the predicted miR-664a-5p binding sites with Punisher. (f, g) qRT-PCR analysis of transcript level of miR-664a-5p after Punisher knockdown or overexpression. (h) The prediction of coding sequence in luciferase constructs with the wild type (Luc-Punisher-wt) and mutated (Luc-Punisher-mut). (i) Luciferase assay of HEK 293 cells cotransfected with a scrambled control, miR-664a-5p mimics, and a luciferase reporter containing Punisher-wt or mutant constructs. (j, k) Pulldown assay with biotinylated miRNA to test the association between Punisher and miR-664a-5p. VSMCs were transfected with biotinylated WT miR-664a-5p (Bio-664-wt) or biotinylated mutant miR-664a-5p with disruption of basepairing between Punisher and miR-664a-5p (Bio-664-mut). A biotinylated miRNA that is not complementary to Punisher was used as a NC (Bio-NC). 24 h after transfection, cells were harvested for biotin-based pulldown assay using streptavidin magnetic beads (Sigma). The bound RNAs were purified using TRIzol for the analysis, and Punisher expression levels were analyzed by qRT-PCR. Data are shown as mean ± s.e.m. of three independent experiments. Scale bars: 20 *μ*m. ^∗^*p* < 0.05; ^∗∗^*p* < 0.01; ^∗∗∗^*p* < 0.001.

Next, we further investigated the potential molecular mechanism through which Punisher regulates VSMC apoptosis. Since Punisher knockdown had slight or no effect on the expression of the nearby coding genes (Figure S5), we ruled out the possibility that Punisher affected the nearby cis-acting genes. Then, we observed Punisher localized in the cytoplasm rather than in the nucleus (Figures 3(a) and 3(b)). Two mechanisms of action of lncRNA in the cytoplasm have been identified so far: lncRNA might act as competing endogenous RNA, sequestering miRNAs to restore mRNA translation; they might promote STAU1-mediated mRNA decay via ALU elements [38, 39]. Bioinformatics analysis showed that there was no ALU element in the sequence of Punisher (http://www.repeatmasker.org). Additionally, RIP results indicated that Punisher can interact with the protein AGO2, a key component of the RISC complex (Figure 3(c)). Therefore, we hypothesized that cytoplasmic Punisher might play a role as a competitive ceRNA.

Next, we examined whether Punisher can interact with miRNAs. We used the bioinformatics program RNAhybrid (https://bibiserv.cebitec.uni-bielefeld.de/rnahybrid) to predict which miRNAs can bind to Punisher and noticed that Punisher contains binding sites for various miRNAs, including miR-134-3p, miR-194-3p, miR-378a-5p, miR-615-5p, miR-26b-3p, miR-365a-5p, and miR-664a-5p (Figure S6). Among of them, we noticed that Punisher contains a potential binding site of miR-664a-5p (Figures 3(d) and 3(e)), while it was subsequently approved to be negatively regulated by knockdown or overexpression of Punisher (Figures 3(f) and 3(g), Supplementary Fig 5c-d), the knockdown and overexpression efficiency was firstly detected by qPCR (Figure S6a-b). Therefore, we generated luciferase constructs with the wild type (Luc-Punisher-wt) and mutated (Luc-Punisher-mut) Punisher sequence upstream the luciferase coding sequence (Figure 3(h)) and performed luciferase assays. We found that miR-664a-5p decreased the luciferase activity of Punisher wild type (Figure 3(i)). The luciferase-mutant Punisher construct also leads to a small, yet significant reduction of luciferase activity, but much lower than wild type. These evidences suggested that Punisher can directly interact with miR-664a-5p.

To further demonstrate that miR-664a-5p binds to Punisher, we performed biotin–avidin pulldown assays as previously described [40]. VSMCs were transfected with biotinylated miR-664a-5p (WT or mutant) for 24 hours and then harvested for biotin-based pulldown assay [41]. Punisher was pulled down and evaluated by qPCR. We observed that Punisher was pulled down by wide-type miR-664a-5p, whereas the mutated miR-664a-5p that disrupts basepairing of the putative Punisher binding site did not display the binding activity with Punisher (Figures 3(j) and 3(k)), implying that miR-664a-5p could recognize Punisher in a sequence-specific manner.

### 2.4. miR-664a-5p Regulates Apoptosis and H_2_O_2_ Mediated Mitochondrial Fission in VSMCs

**Figure 4:**
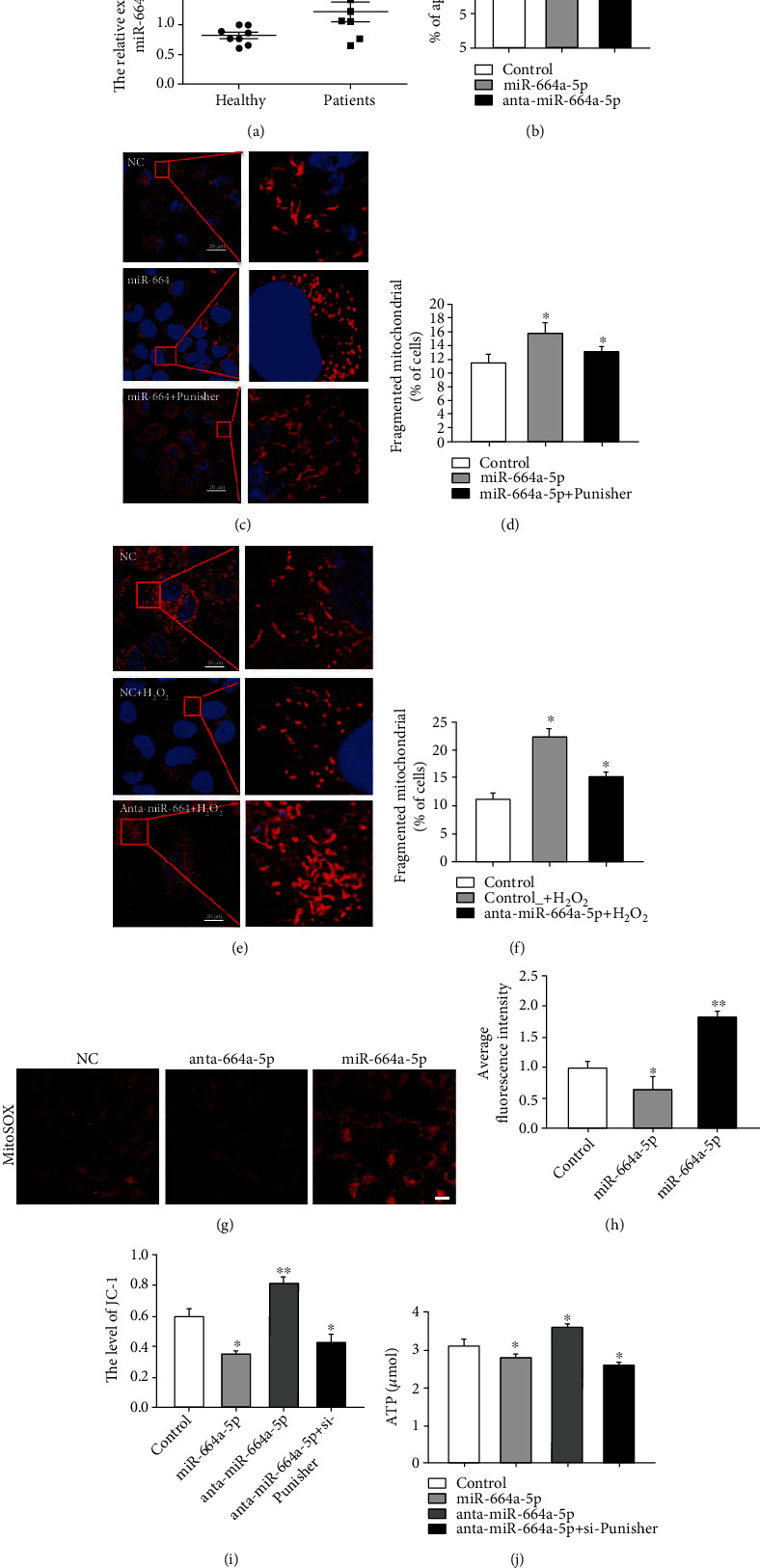
miR-664a-5p regulates biological function in VSMCs through targeting OPA1. (a) qRT-PCR analysis of miR-664a-5p mRNA expression in atherosclerotic plaques of patients and healthy people. (b) Annexin V-FITC/propidium iodide staining and FACS quantification of the number of apoptotic cells in HA-VSMC after transfection of miR-664a-5p mimic. (c, d) MitoTracker staining of mitochondrial fission in VSMC after transfection of miR-664a mimic or Punisher. (e, f) MitoTracker staining of mitochondrial fission in VSMCs after transfection of miR-664a inhibitor with or without H_2_O_2_ stimulation. (g, h) Evaluation of ROS production after transfection of miR-664a-5p mimic or inhibitor. (i) Measurement of mitochondrial membrane potential after transfection of miR-664a-5p inhibitor or Punisher. (j) Evaluation of ATP production after transfection of miR-664a-5p inhibitor or Punisher. All values are the average of at least three biological replicates, and data shown are the mean ± SD. Scale bars: 20 *μ*m. ^∗^*p* < 0.05; ^∗∗^*p* < 0.01.

Given the direct interaction between Punisher and *miR-664a-5p*, we hypothesized that *miR-664a-5p* might play a vital role in AS. To this end, we detected the expression of miR-664a-5p in the aortic plaques of atherosclerotic patients. The results revealed that miR-664a-5p expression was upregulated in patients compared with the healthy people (Figure 4(a)), indicating that miR-664a-5p might be a significant regulator in AS. Therefore, we investigated the functional role of miR-664a-5p in VSMCs. Firstly, we explored the effect of miR-664a-5p on apoptosis by Annexin V-FITC/PI staining. We observed that miR-664a-5p overexpression significantly induced apoptosis in VSMCs and decreased after transfection of miR-664a-5p inhibitors (Figure 4(b)). Most importantly, upregulation of miR-664a-5p clearly promoted mitochondrial fission while cotransfection with Punisher rescued this result (Figures 4(c) and 4(d)). The evidence was also observed in H_2_O_2_ treatment, and knockdown of miR-664 attenuated mitochondrial fission induced by H_2_O_2_ (Figures 4(e) and 4(f)). In mitochondrial function analysis, upregulation of miR-664 increased ROS production (Figures 4(g) and 4(h)). Meanwhile, the mitochondrial membrane potential (Figure 4(i)) and the production of ATP (Figure 4(j)) were also abated by miR-664 overexpression, as well as increase by miR-664 inhibitor transfection, whereas cotransfection of si-Punisher recovered this effect. These results indicated that miR-664a-5p is critically involved in the regulation of apoptosis and mitochondrial dynamics in VSMCs, which could be mediated by Punisher.

### 2.5. miR-664a-5p Participates in the Regulation of OPA1 Expression in VSMCs

**Figure 5:**
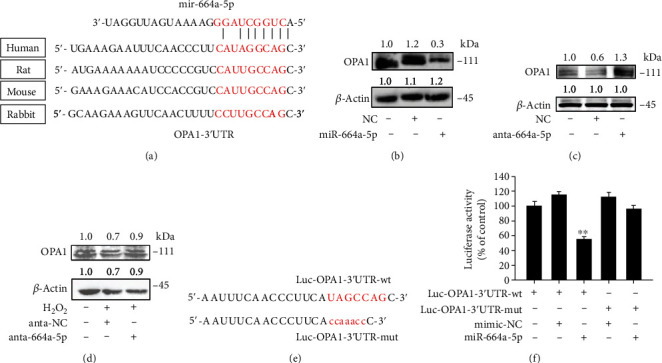
miR-664a-5p participates in the regulation of OPA1 expression in VSMCs. (a) Putative miR-664a-5p-binding site in the 3′UTR region of OPA1 in different species. (b–d) OPA1 expression levels were regulated by miR-664a-5p, analyzed by western blotting after transfection of miR-664 a-5p mimic or inhibitor with or without 100 *μ*M H_2_O_2_ induction. (e) Construction of OPA1-WT and mutant luciferase report plasmid. (f) HEK 293 cells cotransfected with the luciferase constructs of OPA1-WT or mutant, along with the expression plasmids for miR-664a-5p or negative control, and the luciferase activity was analyzed. Data are shown as mean ± s.e.m. of three independent experiments. Scale bars: 20 *μ*m. ^∗^*p* < 0.05.

miRNAs negatively regulate gene expression by promoting mRNA degradation or suppressing mRNA translation. To explore the underlying mechanism of miR-664a-5p, we screened the target genes of miR-664a-5p and identified those involved in cell apoptosis and mitochondrial function using the miRBase (http://www.mirbase.org) and TargetScanHuman databases (http://www.targetscan.org). By analyzing the 3′UTR sequence of the putative targets, we found that OPA1 exhibited the best predicted alignment; notably, the alignment was conserved in several species (Figure 5(a)). Therefore, we detected whether dysregulation of miR-664a-5p could affect the expression of OPA1. Overexpression of miR-664a-5p resulted in a strong reduction of OPA1 levels (Figure 5(b)), while inhibition of miR-664a-5p led to the obvious increase of OPA1 levels with or without H_2_O_2_ stimulation (Figures 5(c) and 5(d)).

To further verify the interaction between miR-664-5p and OPA1 3′UTR, we performed luciferase gene reporter assays and investigated whether miR-664a-5p can regulate the translation of OPA1. We constructed luciferase reporter plasmids in which the WT (Luc-OPA1-3′UTR) or the mutant (Luc-OPA1-3′UTR-mut) 3′UTR of OPA1 was upstream of the luciferase coding sequence (Figure 5(e)). We found that miR-664a-5p suppressed the expression of OPA1, but not that of the mutant (Figure 5(f)). Collectively, these data indicated that OPA1 is the direct target of miR-664a-5p.

### 2.6. OPA1 Regulates Cell Apoptosis and Mitochondrial Fusion

**Figure 6:**
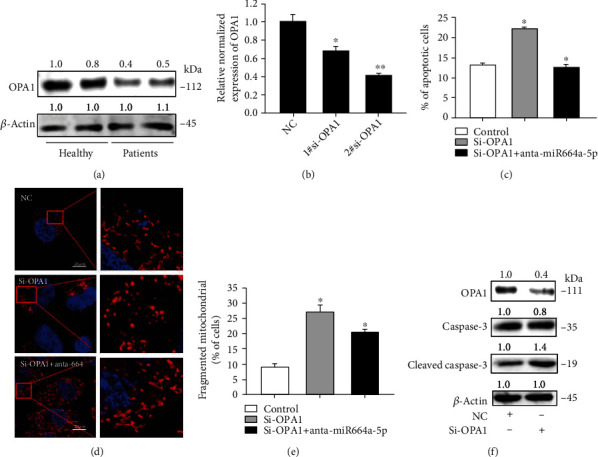
OPA1 is able to regulate cell apoptosis and mitochondrial fission in VSMCs. (a) Detection of OPA1 expression in atherosclerotic plaques of patients and healthy people by western blotting. (b) Knockdown efficiency of OPA1 siRNAs after 24 hours transfection in VSMCs. (c) Annexin V-FITC/propidium iodide staining and FACS quantification of the number of apoptotic cells after transfection of OPA1 siRNA or miR-664a-5p inhibitor. (d, e) MitoTracker staining of mitochondrial fission in VSMCs after transfection of OPA1 siRNA or miR-664 inhibitor. (f) The protein levels of apoptotis markers were determined by western blotting. All values are the average of at least 3 biological replicates, and data shown are mean ± SD. Scale bars: 20 *μ*m. ^∗^*p* < 0.05.

OPA1 is mainly localized in the mitochondrial intermembrane space and serves as a significant regulator in mitochondrial fusion. Recent studies suggested that OPA1 participates in the development of cardiovascular diseases [42, 43]. However, the functional role of OPA1 in AS and underlying molecular mechanisms are still largely unknown. To investigate OPA1 function, we first detected the expression level of OPA1 in patient arteries and observed that the quantity of OPA1 was clearly downregulated compared with healthy people (Figure 6(a)). Then, we knocked down *OPA1* expression by RNA interference. Of the siRNA tested, si-OPA1 #2 was the most effective and therefore used for the subsequent experiments (Figure 6(b)). We next detected knockdown of *OPA1* expression resulted in increase of apoptosis (Figure 6(c)) and mitochondrial fission (Figures 6(d) and 6(e)), whereas cotransfection of si-OPA1 and anti-miR-664a rescued these effect. Additionally, downregulation of *OPA1* increased active caspase-3 levels (Figure 6(f)). The evidences supported that OPA1 inhibits apoptosis and mitochondrial fission in VSMCs which could be negatively regulated by miR-664a-5p.

### 2.7. GapmeR-Punisher Regulates Apoptosis and Mitochondrial Fusion by Targeting miR-664a-5p and OPA1 in VSMCs

**Figure 7:**
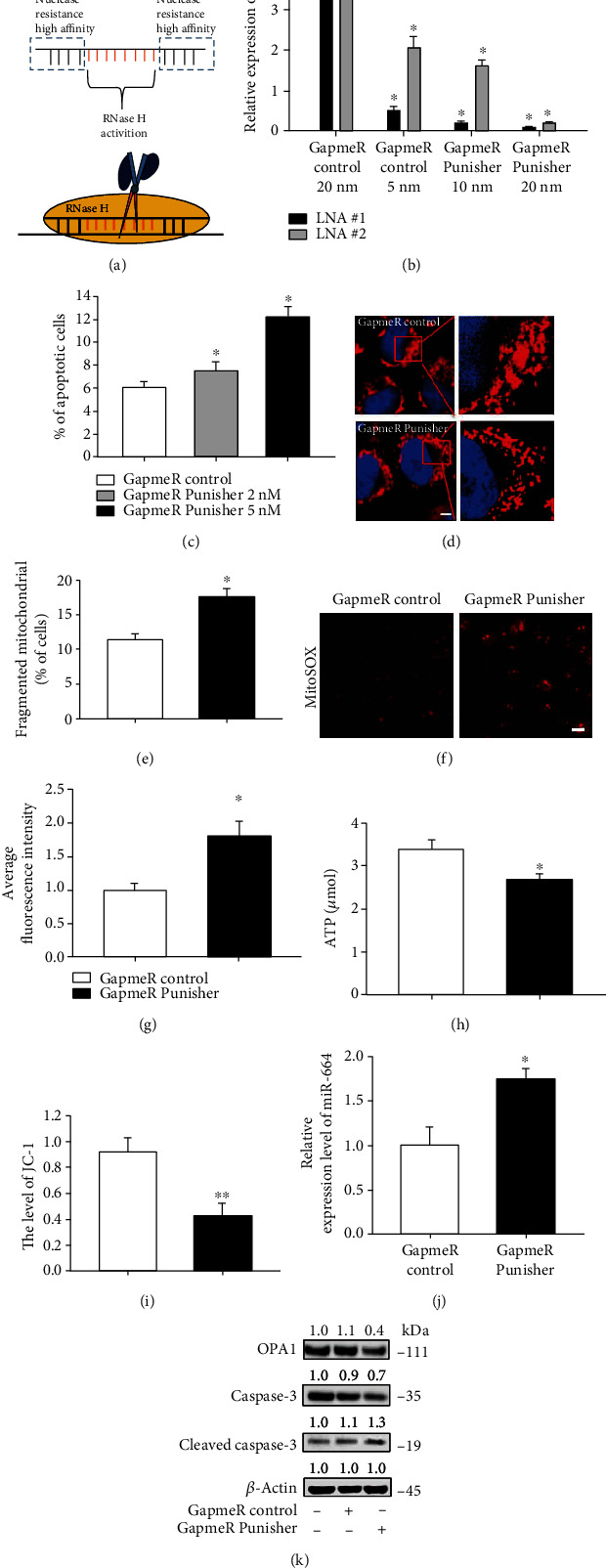
GapmeR-Punisher-mediated silencing induces cell apoptosis and mitochondrial fission in VSMCs. (a) The diagram of design and principle of GapmeR-Punisher. (b) qRT-PCR analysis of knockdown efficiency after transfection of different doses of GapmeR-Punisher. (c) Annexin V-FITC/propidium iodide staining and FACS quantification of the number of apoptotic cells after transfection of GapmeR-Punisher. (d, e) Detection of mitochondrial fission through MitoTracker staining in VSMC after LNA-Punisher transfection. (f, g) Evaluation of ROS production after transfection of LNA-Punisher. (h) Evaluation of ATP production after transfection of LNA-Punisher. (i) Measurement of mitochondrial membrane potential after LNA-Punisher transfection by detecting JC-1 expression using ELISA. (j) qRT-PCR analysis of miR-664a-5p mRNA expression level after transfection of GapmeR-Punisher. (k) The apoptosis proteins were determined by western blotting after transfection of GapmeR-Punisher. All values are the average of at least 3 biological replicates, and data shown are mean ± SD. Scale bars: 20 *μ*m. ^∗^*p* < 0.05; ^∗∗^*p* < 0.01.

To further verify the functional role of Punisher, we used LNA-GapmeR ASOs, which can trigger RNAse-H-dependent degradation of target genes, to efficiently knock down Punisher expression (Figure 7(a)). Two GapmeR DNA antisense oligos targeting Punisher were designed and constructed by Exiqon (Denmark) and used to perform loss-of-function studies in VSMCs. qRT-PCR analysis showed that transfection of two different LNA-GapmeRs targeting Punisher (LNA#1 and LNA#2) obviously attenuated Punisher expression (Figure 7(b)). LNA#1 was selected for the further experiments due its more effective inhibition. Meanwhile, we also observed the increase of apoptosis (Figure 7(c)), mitochondrial fission (Figures 7(d) and 7(e)), and ROS production (Figures 7(f) and 7(g)) as well as downregulation of mitochondrial function, including ATP production (Figure 7(h)) and mitochondrial membrane potential (Figure 7(i)). Transfection of LNA-Punisher also enhanced miR-664a-5p expression (Figure 7(j)). Furthermore, downregulation of Punisher suppressed OPA1 expression and promoted cleaved caspase-3 activity, which resulted in cell apoptosis (Figure 7(k)). Taken together, these results provided that Punisher negatively regulates cell death and mitochondrial fission in VSMCs.

### 2.8. Overexpression of Punisher Attenuated Neointima Formation Induced in a Balloon-Injured Carotid Artery Model

**Figure 8:**
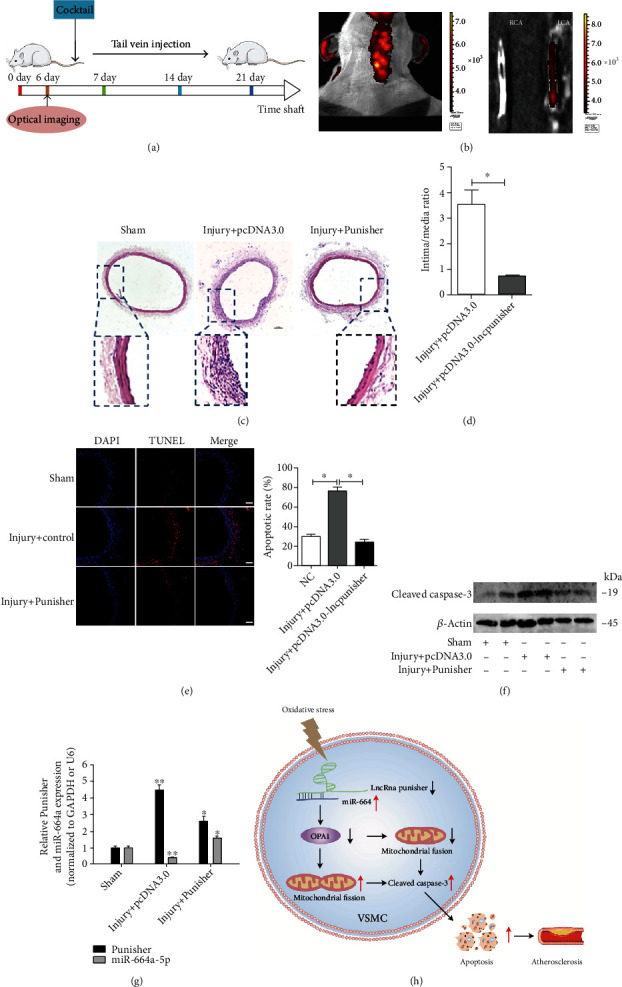
Effect of the Punisher on neointimal hyperplasia in a rat carotid artery balloon injury model. (a) Systematic administration of cocktail in rat carotid balloon injury model. (b) The in vivo bioluminescence images of the distribution of Cy5.5-labeled Punisher/PEI/PEG complexes in the carotid balloon injury model. (c) Punisher suppressed neointimal hyperplasia in injured arteries as determined by H&E staining. (d) Morphometric analysis of the intima/media (I/M) area ratio. (e, f) Enforcement of Punisher distinctly attenuated apoptosis of injured arteries in vivo determining by immunofluorescence and western blotting. (g) Transfection of the Punisher remarkably restrained the expression of miR-664a-5p level in vivo as determined by qRT-PCR compared with the uninjured group. (h) The graphic illustration of Punisher-miR-664a-5p–OPA1 signaling axis in VSMCs. Results were quantified using the ImageJ software. Data are shown as mean ± SD. Scale bar, 100 mm. *n* = 6 for (b–d). ^∗^*p* < 0.05; ^∗∗^*p* < 0.01.

To determine the therapeutic effects of Punisher in in vivo, we used the carotid balloon injury model in rat, and enforced Punisher expression using a plasmid/polyethylenimine (PEI)/polyethylene glycol (PEG) method to regulate Punisher level. We injected these pcDNA3.0-Punisher or pcDNA 3.0 cocktails via the tail vein after the balloon injury following the schematic diagram in Figure 8(a). Firstly, we confirmed the enrichment of Punisher-Cy5.5 in injured lesion after 6 hours injection via the IVIS spectrum in vivo imaging system, and we observed a remarkable accumulation of Punisher in neointima and media of injury vessels compared with control (Figure 8(b)). Encouragingly, the symptoms were significantly alleviated after Punisher upregulation (Figure 8(c)), and the analysis of intima/media ratio showed a 70% reduction from the vehicle-treated group to the Punisher-treated group (*p* < 0.05, Figure 8(d)), indicating a protective role for Punisher in alleviating intimal hyperplasia. Concomitantly, we found that there was distinct apoptosis in vessel lesion, while Punisher treatment significantly suppressed this effect (Figure 8(e)). Besides, western blotting result showed that active caspase-3 expression was suppressed in Punisher-infected balloon-injured arteries compared with the control group (Figure 8(f)), and this increase was accompanied by a marked decrease of miR-664a-5p expression and upregulation of Punisher (Figure 8(g)). These data supported that Punisher serves as a significant regulator in mitochondrial fusion and neointimal hyperplasia in vivo.

## 3. Discussion

Abnormal apoptosis and proliferation of VSMCs are critically involved in AS. Several studies have shown that in the early stages of AS, a series of pathological phenomena take place, including the increase in the number of vascular cells, the thickening of the intima, and the narrowing of the lumen. While in the late stages of atherosclerosis, excessive VSMC apoptosis contributed to the instability of the plaque and increase of thrombosis, as well as promotion of calcification and atherosclerotic stenosis [44, 45]. Therefore, the targeting of dysregulated VSMCs may be important in the treatment of AS.

In this study, we identified Punisher as a key regulator of cell apoptosis and mitochondrial dynamics which finally contributes to the progression of atherosclerosis. We found that Punisher could only regulate proliferation and migration slightly in VSMCs, whereas it strongly suppressed cell apoptosis as well as mitochondrial dynamics. Mechanistically, we found that Punisher directly binds to miR-664a-5p, leading to degradation of the mitochondrial fusion protein OPA1, which results in dysfunction of mitochondria. Taken these interactive pathways into consideration, we proposed a putative mode for the actions of Punisher (Figure 8(h)). These results are of great interest because they provide novel evidence for the biological and clinical significance of Punisher expression as a potential biomarker for patients with AS and as a potential therapeutic target to treat human cardiovascular diseases.

Oxidative stress and apoptosis are important mechanisms that promote the development of AS [46–48]. Reactive oxygen species (ROS) are major regulators of various signal transduction systems in atherosclerotic vasculitis, which is associated with the formation of lipid stripes, progression of lesions, and final plaque rupture [49]. The mitochondrial respiratory chain is the main source of ROS. Mitochondrial dysfunction can affect AS progression through oxidative stress, inflammation, apoptosis, and cholesterol accumulation. OPA1 is a nuclear-encoded mitochondrial protein. OPA1 can be alternatively spliced, forming multiple subtypes, which play an important role in mitochondrial morphology and structure. Recent studies have shown that OPA1 participates in mitochondria-dependent apoptosis in cardiomyocytes and neuronal cells [50, 51]. However, to date, it has not been reported whether OPA1 can regulate apoptosis in VSMCs. Therefore, we investigated the functional role of OPA1 in VSMCs and found that knockdown of OPA1 promotes cell apoptosis and mitochondrial fission in VSMC. Importantly, we found OPA1 was downregulated in atherosclerotic arteries which finally results in regulating ATP production, ROS release, and mitochondrial membrane potential. Moreover, these evidences could be obviously regulated by Punisher and miR-664a-5p.

lncRNAs are a class of RNA with transcripts longer than 200 nucleotides and are generally considered not to encode proteins. At first, they were considered as the by-products of RNA polymerase 2 transcription and therefore a “noise” in genomic transcription and with no biological functions. Recently, however, many studies have shown that lncRNAs are crucially involved in various processes, such as chromatin modification, transcriptional activation, transcriptional interference, nuclear transport, and genomic imprinting. Additionally, functional studies have revealed that lncRNAs are important regulatory molecules involved in the development of cardiovascular diseases by regulating the proliferation, apoptosis, injury, autophagy, and differentiation of a variety of cells [52–56]. However, the functional role and underlying molecular mechanisms of lncRNA in the mitochondrial dynamics of VSMC are largely unknown. This study shows that the lncRNA Punisher acts as an endogenous “sponge” of miR-664a-5p, thereby regulates the translational repression on *OPA1* which is vitally involved in mitochondrial homeostasis and cell apoptosis in VSMCs.

Furthermore, to efficiently knock down Punisher, we used LNA-GapmeR ASOs, single-stranded gene silencing oligonucleotides that seem to be more efficient than siRNA in decreasing the activity of lncRNA [57, 58] (Li, 2022 #3335). LNA-GapmeR ASOs contain mainly two parts: the central phosphorothioate DNA gap that binds the RNA target and elicits RNase H-dependent degradation and the “wings,” on both sides, which consist of sugar-modified nucleotides such as 2′-O-methoxyethyl-RNA (2′-*O*-MOE-RNA) or LNA, which enhance binding affinity and nuclease stability [58]. LNA-ASOs are becoming a potential and attractive therapeutic modality to target undruggable pathways ENREF_32 [59, 60]. In this light, we designed and synthesized two LNA-GapmeRs targeting Punisher and showed their striking knockdown effect. The delivery of Punisher-targeting LNA-GapmeRs triggered apoptosis and mitochondrial fission in VSMCs, confirming that ASO-based gene silencing of Punisher could be a useful tool for AS research and therapy. In addition, there are a variety of nucleic acid delivery methods, such as nanomedicines and exosomes [61–63].

In summary, our data provided evidence that Punisher exerts an antiapoptotic and antimitochondrial fission function in VSMCs by targeting miR-664a-5p and its downstream target OPA1 and therefore alleviating the development of atherosclerosis. This discovery may shed new light to understand the complex molecular mechanisms regulating the mitochondrial network and suggest the possible targeting of Punisher in AS therapy.

## 4. Materials and Methods

### 4.1. Patient Samples

Tissue samples from patients with AS and healthy subjects were collected at the Affiliated Cardiovascular Hospital of Qingdao University in Qingdao (China) from March 2017 to July 2019. The study group consisted of 12 patients scheduled for Coronary Artery Bypass Graft (CABG) surgery at the Hospital of Qingdao University. Inclusion criteria were patients with severe 3-vessel disease confirmed by coronary angiography and requiring CABG surgery. Exclusion criteria were cardiomyopathy, liver or kidney dysfunction, infection, cancer, autoimmune disease, and sepsis. The control group consisted of 10 healthy people who showed no clinically obvious blocking of the coronary arteries. Tissue specimens were rapidly frozen in liquid nitrogen prior to analysis. The research was approved by the Institutional Review Boards of Qingdao University Health Science Center. Informed consent was obtained from all participants prior to study participation. The details of all samples are in Table S1.

### 4.2. Cell Culture and Treatment

The human aorta VSMC line HA-VSMC was obtained from the Chinese Type Culture Collection (Chinese Academy of Sciences, Shanghai, China) and cultured in Dulbecco's modified Eagle's medium (GIBCO, Grand Island, NY, USA) containing 10% fetal bovine serum (ExCell Bio., Shanghai, China) in a 5% CO_2_ humidified incubator at 37°C. Cells were transfected with Lipofectamine 2000 (Life Technologies, Carlsbad, USA) following the manufacturer's recommendations. The HA-VSMC line was passaged in our laboratory for less than two months and maintained according to the supplier's instructions.

### 4.3. RNA Isolation and Quantitative PCR (qPCR)

Total RNA was isolated using TRIzol reagent (Sigma, St. Louis, MO, USA). The nuclear and cytoplasmic fractions were isolated as previously described [64]. Complementary DNA was synthesized using the SPARKscript II RT kit (With gDNA Eraser) (Sparkjade Science Co., Ltd, Shandong, China), and qPCR assays were carried out using the SYBR Premix Ex Taq (Takara Bio Inc., Dalian, China) and the Mir-X miRNA First-Strand Synthesis Kit (Takara Bio Inc., Dalian, China). Gene expression was measured by qPCR system (Roche, Basel, Switzerland) using SYBR-green (Roche, Basel, Switzerland). Gene expression was normalized to GAPDH or U6 snRNA, respectively. The primers used in the qPCR assays are listed in table S2.

### 4.4. Small Interfering RNA (siRNA) Knockdown

Human si-Punisher and control were designed and constructed by Genepharma (Shanghai, China). Human OPA1 (SR303287) and scrambled (SR30004) siRNAs were purchased from OriGene (Origene Technologies Inc., Rockville, USA). Confluent cultures of HA-VSMC were transfected with siRNAs using Lipofectamine 2000. qRT-PCR measurements of *OPA1* and Punisher expression were performed to assess the knockdown efficiency.

### 4.5. Cell Counting Kit-8 (CCK-8) Assay

The CCK-8 assay (7Sea-Cell Counting Kit, Shanghai, China) was performed to evaluate cell proliferation. The cells (5 × 10^3^) were seeded into 96-well plates and incubated for 12, 24, and 36 hours after transfection. The CCK-8 solution (10 *μ*L) was then added to each well. The cells were incubated at 37°C for three more hours, and the absorbance was measured at 450 nm (Cui, 2021 #3336) in a microtiter plate reader (Bio-Tek, Vermont, USA).

### 4.6. Wound Healing Assay

VSMCs were grown up to 70-80% confluency in 6-well plates and transfected, as indicated. Twelve hours after transfection, when the cells had reached almost 100% confluency, wounds were made using a 1,000 *μ*l disposable pipette tip. The width of the scratch was visualized and photographed immediately and at different time points after wounding using the LSM510 META microscope. The Image J software, version 1.8.0 (NIH, Bethesda, MD, USA), was used for the analysis of the data.

### 4.7. Apoptosis Assay

Cell apoptosis was determined using an Annexin V-FITC/propidium iodide (PI) apoptosis kit (BD, San Jose, CA, USA) by flow cytometry. Treated cells were pelleted by centrifugation at 800 × g for 5 min, washed twice with cold PBS, and resuspended into 400 *μ*l binding buffer. Ten microliters of Annexin V-fluorescein isothiocyanate (FITC) solution and subsequently 5 *μ*L of PI per 100 *μ*L of cell suspension were then added, and the mixture was incubated for 15 min in the dark at room temperature (20-25°C). Finally, the fluorescence of the cells was recorded using the Accuri C6 flow cytometer (BD, USA).

### 4.8. Mitochondrial ROS Assessment

The levels of mitochondrial ROS were detected using the fluorescent probes MitoSOX™ Red (Molecular Probes, Life Technologies, Carlsbad, CA, USA), and images were obtained using a camera connected to a confocal microscope (LSM510 META, Zeiss).

### 4.9. Measurement of Mitochondrial Membrane Potential (MMP) by JC-1

MMP was evaluated using the lipophilic cationic probe 5,5,6′,6′-tetrachloro-1,1′,3,3′-tetraethylbenzimidazolcarbo-cyanine iodide (JC-1) according to a previously described instructions [65]. In brief, VSMC cells were cultured in 24-well plates (5 × 10^5^ cells/mL). After transfection of target genes for 24 hours, detached and mixed 0.5 ml cell suspension with 0.5 ml JC-1(1 *μ*g/ml), incubated for 20 min at 37°C, transferred 100 *μ*l per well into opaque black plate, and immediately analyzed in the microtiter plate reader (Bio-Tek, Vermont, USA).

### 4.10. ATP Concentration

ATP measurement was performed according to the manufacture's guidelines [66]. Briefly, 20 *μ*l of cell lysis solution was mixed with 5 *μ*l of ATP solution for OD reading at 570 nm using the microtiter plate reader (Bio-Tek, Vermont, USA). The amount of ATP production was calculated from the standard curve constructed with 0-100 nmol ATP. Protein concentration was determined by using BCA protein assay kit (Beyotime, Shanghai, China).

### 4.11. Immunoblotting

Immunoblot was performed as previously described [67] with slight modifications. In brief, the treated cells were lysed for 30 min on ice with RIPA buffer (0.5 mM EDTA, 50 mM Tris (pH 7.4), 1% NP-40, 150 mM NaCl, 0.1% SDS, and 0.5% sodium deoxycholate) containing a protease inhibitor cocktail (Roche, Germany). Equal protein loading was achieved by measuring the protein concentration of each sample with a BCA protein assay kit (Solarbio, Beijing, China). The samples were separated by 10-12% SDS polyacrylamide gel electrophoresis (SDS-PAGE) and transferred to polyvinylidene fluoride (PVDF) membranes. Membranes were blocked in 5% nonfat milk for 1 hour at room temperature and probed using the following primary antibodies: anti-OPA1 (abcam157457, Cambridge, MA, USA) purchased from Abcam, and antibodies against p53 (CST9282, Danvers, MA, USA), caspase-3 (CST9662, Danvers, MA, USA), Bcl-2 (CST15071, Danvers, MA, USA), and *β*-actin (CST4967, Danvers, MA, USA) obtained from Cell signaling Technology. After three washing, the membranes were incubated with horseradish peroxidase-conjugated secondary antibodies (CST7074, Boston, MA, USA) for 1 h. Signals were visualized using the enhanced chemiluminescence imaging system (Millipore, USA). The protein levels were analyzed using the Image J software and normalized to the corresponding *β*-actin levels.

### 4.12. Subcellular Fractionation

Subcellular fractions were extracted as described before [33]. The cells were collected and washed twice with cold PBS and briefly centrifuged at 12,000 × g at 4°C. The pellets were resuspended in 0.2 mL of isolation buffer A (1 mM EDTA, 1 mM EGTA, 20 mM HEPES (pH 7.5), 1.5 mM MgCl_2_, 10 mM KCl, 1 mM DTT, 0.1 mM phenylmethanesulfonyl fluoride (PMSF), and 250 mM sucrose) containing a protease inhibitor cocktail, then homogenized, and centrifuged at 12,000 rpm for 5 min at 4°C to isolate nuclei and debris. Meanwhile, the mitochondria- enriched heavy membrane pellets were obtained from the supernatants by centrifugation at 12,000 rpm for 10 min at 4°C.

### 4.13. Pulldown Assay with Biotinylated MicroRNA (miRNA)

Pulldown assays were performed as described earlier [33] with a few modifications. HA-VSMC (5′ 10^6^cells/well) were transfected with biotinylated *miR-664a-5p* wild type (WT) and mutant (50 nM) for 24 h. The treated cells were washed with cold PBS and resuspended in lysis buffer with 0.05% Igepal and 60 U mL/L Superase-In (Ambion, Waltham, MA, USA) for 10 min on ice. The lysates were subsequently incubated with M-280 streptavidin magnetic beads (Sigma, St. Louis, MO, USA) preincubated with bovine serum albumin (BSA; Millipore) and yeast tRNA (Sigma) to block nonspecific binding sites. The beads were then incubated for 4 h at 4°C rocking gently and washed five times with a low salt buffer (20 mM Tris-HCl (pH 8.0), 2 mM EDTA, 0.1% SDS, 150 mM NaCl, and 1% Triton X-100) and once with a high salt buffer (20 mM Tris-HCl (pH 8.0), 0.1% SDS, 2 mM EDTA, 500 mM NaCl, and 1% Triton X-100). The bound RNAs were purified using TRIzol.

### 4.14. Microarray Analysis

The Human Transcriptome Array 2.0 (HTA 2.0; Affymetrix, Santa Clara, CA) was performed in this study [68]. HTA 2.0 covers global profiling of full-length transcripts, containing more than 40,000 noncoding and 245,000 coding transcripts in human genome. Each transcript is accurately identified by probes target specific exons or splice junctions. Total RNAs were prepared from VSMC cells, with three biological replicates for each group. The differentially expressed lncRNAs with statistical significance were identified through volcano plot filtering, with a predefined threshold (fold change ≥ 1.5 and *p* value < 0.05). The cDNA labeling, microarray hybridization and bioinformatics analysis were performed by the National Engineering Center for Biochip (Shanghai, China).

### 4.15. Luciferase Assays with OPA1 3′ Untranslated Region (3′UTR), miR-664a-5p, and Punisher

The (3′UTR) of *OPA1* was amplified by PCR using the primers 5′-ATCAATTT CAACCCTTCATAGCCAGCA-3′ and 5′-CTAGTGCTGGCTATGAAGGGTTG AAATTGAT-3′. The QuikChange II XL Site-Directed Mutagenesis Kit (Stratagene, Agilent Technology, Palo Alto, USA) was used to generate a mutated *OPA1* 3′UTR. WT and mutated 3′UTR were subcloned into the pGL3 vector (Promega, Madison, Wisconsin, USA), immediately downstream the coding region of the luciferase gene. Similarly, the Punisher WT and the mutant sequences were subcloned in the luciferase reporter vector. The primers used to amplify Punisher were 5′-ATCCAGC ACCAGGGTCAGCTCCCGTGGCCAGTCA-3′ and 5′-CTAGTAGGTCGTGGTC CCAGTCGAGGGCACCGGTCAGT-3′. The *miR-664a-5p* reporter was constructed according to the method previously described [24, 69]. Transfection of the construct, as indicated, was performed using Lipofectamine 2000 (Thermo Fisher Scientific, USA).

### 4.16. RNA Immunoprecipitation (RIP)

RIP assays were carried out to identify regions of the genome with RNA-binding proteins. VSMCs were lysed in RIPA buffer containing 0.1 mM PMSF and 1% protease inhibitor cocktail on ice. After 10 min incubation, the cells were centrifuged at 12,000 rpm for 20 min at 4°C. Five hundred micrograms of cell lysates was then incubated with the indicated primary antibody at 4°C overnight with gentle rocking. Protein A/G-agarose beads (Thermo Fisher Scientific, USA) were added, and the mixtures were incubated for 4 hours at 4°C with shaking. After washing, immunoprecipitated RNA-binding proteins and their bound RNA were isolated. qRT-PCR assays were then performed.

### 4.17. Fluorescence In Situ Hybridization (FISH)

FISH assays were performed according to a previously described method [70]. The Punisher detection probe (Takara, Bio Inc., China) was used. The sequence of the Punisher probe for FISH was Digoxin-5′-CATCGAGTGTTCTGGCATCCACCGCAACCT-3′-Digoxin. The Cy5-labeled Punisher and FISH Kit (RiboBio, Guangzhou, China) were used according to the manufacturer's guidelines. Images were taken using a confocal microscope (LSM510 META, Zeiss).

### 4.18. Confocal Imaging

VSMCs were stained with MitoTracker Red CMXRos (Molecular Probes) to visualize mitochondrial fission according to manufactures instructions [71]. In short, cells were plated onto coverslips coated with 0.01% poly-L-lysine. After treatment, the cells were stained for 20 min at 37°C with 0.02 mM MitoTracker Red probe and second antibody and then with DAPI for 10 min. Mitochondria were imaged using the confocal microscope. Six distinct fields for each 50 cells were counted. The percentage of cells with fragmented mitochondria relative to the total number of cells is presented as the mean ± s.e.m. of at least three independent experiments.

### 4.19. Histology

Aortic tissues were fixed in 4% paraformaldehyde for 24 h and embedded in paraffin. Serial cryosections of the ascending aorta (on the short axis), 4 *μ*m thick, were selected and stained with hematoxylin and eosin (H&E). Images were captured at 40 magnification. The results are expressed as the mean ± s.e.m. of at least three independent experiments.

### 4.20. Immunohistochemistry

Immunohistochemistry was performed as previously described [72, 73]. In brief, frozen sections were fixed in a methanol/acetone mixture (1 : 1) for 10 min and blocked with 10% (v/v) donkey serum in PBS at room temperature. The slides were then hybridized overnight with primary antibody or normal IgG antibody in 10% horse serum at 4°C, washed gently with PBS, and hybridized with secondary antibody conjugated to horseradish peroxidase (SC2357, Santa Cruz Biotechnology, USA) for 2 h at room temperature. The immunocomplexes were visualized using a DAB solution (Vector Laboratories, Burlingame, CA, USA). The sections were then dehydrated in a series of graded ethanol and xylene, and slides were mounted with the Cytoseal XYL mounting media (Richard-Allan Scientific, Kalamazoo, MI, USA). Finally, the sections were observed and micrographs taken with an optical microscope (Zeiss Axio Imager A2) at 10x or 40x magnification.

### 4.21. Rat Carotid Artery Injury Mode

Male Sprague-Dawley rats weighing 300~350 g were obtained from Vital River Laboratory Animal Technology Co., Ltd (Beijing, China), and animal protocols were approved by the Animal Care and Use Committee of Qingdao University. Balloon injury of the carotid artery model was conducted as previously described [52, 74]. In brief, rats were anesthetized by intraperitoneal administration of pentobarbital (50 mg/kg), and a 2F Fogarty catheter (Edwards Life Sciences LLC Co.) was introduced into the common carotid artery to cause vascular injury. The balloon was inflated to 4 atm of pressure and withdrawn three times to create a 10 mm neointima injury. Following balloon injury, the solution of pcDNA3.0-Punisher/PEI/PEG cocktail (5 mg/kg) was injected into the tail vein after the balloon injury every 7 days for a total of 21 days following the protocol reported before [52], saline or pcDNA3.0 vector cocktail was introduced as control. After 21 days, the rats were euthanized, and the injured and left uninjured common carotid artery were, respectively, removed, fixed, and embedded or stored for RNA and protein extraction.

### 4.22. Statistical Analysis

Data are expressed as the mean ± s.e.m. of at least three independent experiments and evaluated with the Student's *t*-test. Additionally, we used a one-way analysis of variance for multiple comparisons. A value of *p* < 0.05 was considered significant.

## 5. Limitations of the Study

Atherosclerosis is a multifactorial disease involving various cells. The study design did not explore the regulatory roles of Punisher on other vessel cells, such as endothelia cells and macrophages which also contain certain quantity of Punisher. Furthermore, our investigations focused on the significant effect of Punisher on VSMC apoptosis, yet owing to the complex nature of atherosclerosis, other factors are also likely to contribute.

## Data Availability

The data of this study are available from the corresponding author upon reasonable request.

## Supplementary Materials

Supplementary Materials Figure S1: evaluation of Punisher expression in situ in the vessels of patients. Figure S2: Punisher partially regulates cell proliferation and migration of VSMCs. Figure S3: VSMC cells can be induced apoptosis and mitochondrial fission upon H_2_O_2_ treatment. Figure S4: Punisher can regulate antioxidant enzymes. Figure S5: Punisher expression has no relationship with its nearby coding genes. Figure S6: the regulatory effect of Punisher to predicted downstream miRNAs. Table S1: baseline characteristics of the CAD patients receiving CABG surgery and healthy subjects. Table S1: PCR primers used in this study.
