# Supplementary material for: The lncRNA Punisher Regulates Apoptosis and Mitochondrial Homeostasis of Vascular Smooth Muscle Cells via Targeting miR-664a-5p and OPA1
Source: Oxid Med Cell Longev. 2022 May 25;2022:5477024. doi: 10.1155/2022/5477024 (PMC9159832; doi:10.1155/2022/5477024)
Supplement: Supplementary Materials — Figure S1: evaluation of Punisher expression in situ in the vessels of patients. Figure S2: Punisher partially regulates cell proliferation and migration of VSMCs. Figure S3: VSMC cells can be induced apoptosis and mitochondrial fission upon H2O2 treatment. Figure S4: Punisher can regulate antioxidant enzymes. Figure S5: Punisher expression has no relationship with its nearby coding genes. Figure S6: the regulatory effect of Punisher to predicted downstream miRNAs. Table S1: baseline characteristics of the CAD patients receiving CABG surgery and healthy subjects. Table S1: PCR primers used in this study. [file 5477024.f1.pdf]

Supplemental information

The lncRNA Punisher regulates apoptosis and mitochondrial homeostasis of vascular smooth muscle cells via targeting miR-664a-5p and OPA1

Yanyan Yang, Min Li, Yan Liu, Zhibin Wang, Xiuxiu Fu, Xingqiang He, Qi Wang, Xiao-xin Li, Huibo Ma, Kun Wang, Lu Zou, Jian-xun Wang, Tao Yu

SUPPLEMENTAL FIGURES

Figure S1

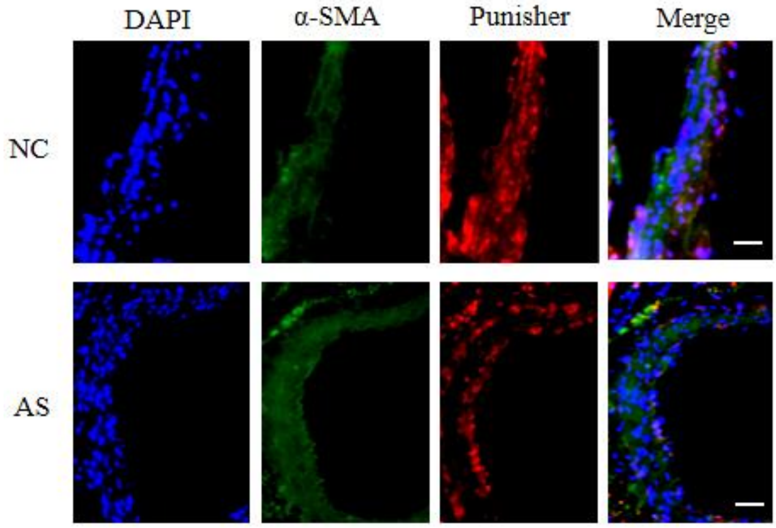

Figure S1. Evaluation of Punisher expression in situ in the vessels of patients. Immunofluorescent staining was used to detect media of aortas (α-SMA) and Punisher expression. Nuclei were stained with DAPI (blue). Scale bars: 20 μm.

Figure S2

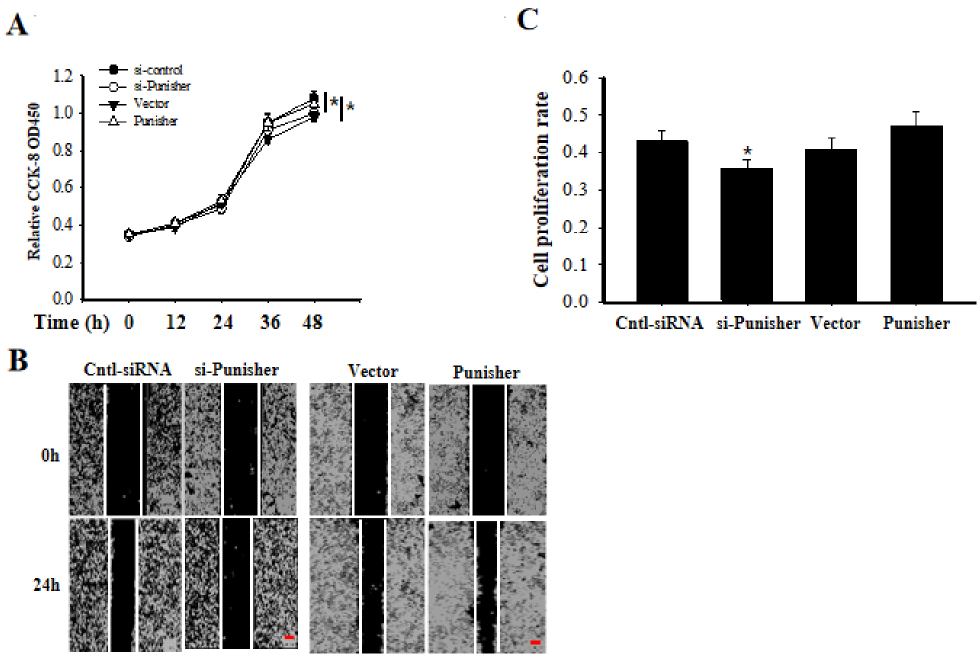

Figure S2. Punisher partially regulates cell proliferation and migration of VSMCs. (A) CCK-8 assay in VSMC transfected with cntl-siRNA, Punisher siRNA, Vector and Punisher, respectively. (B) Evaluation of cell migration via would healing assay after transfection of cntl-siRNA, Punisher siRNA, Vector and Punisher. (C). The cell proliferation rate was analyzed by the Image J software, version 1.8.0 (NIH, Bethesda, MD, USA). All values are the average of at least 3 biological replicates, and data shown are the mean±SD. Scale bars: 20 µm. *P<0.05; **P<0.01.

Figure S3

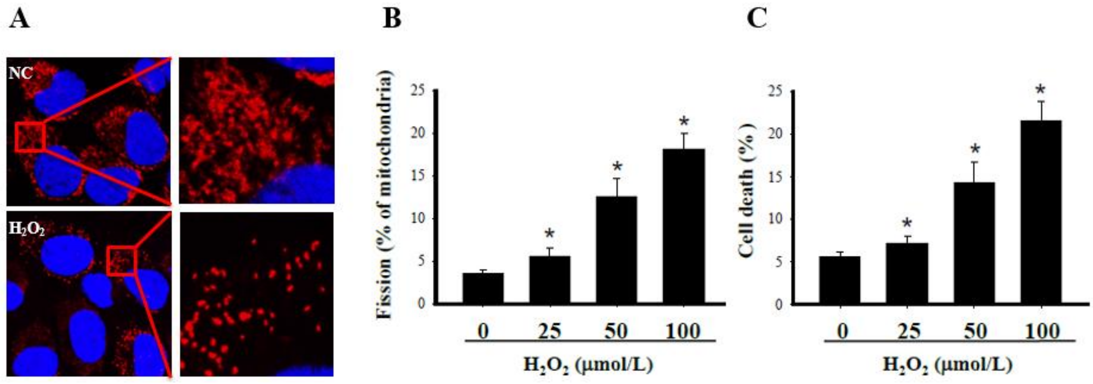

Figure S3. VSMC cells can be induced apoptosis and mitochondrial fission upon H_2O_2 treatment. (A) Mitotracker staining of mitochondrial fission in VSMC after 100 μM H_2O_2 stimulation with 8 hours. (B) Cell number with mitochondrial fission and fission ratio was calculated rate. (C) Cell death was detected with MTT assay after 100 μM H_2O_2 stimulation with different times. All values are the average of at least 3 biological replicates, and data shown are the mean±SD. *P<0.05.

Figure S4

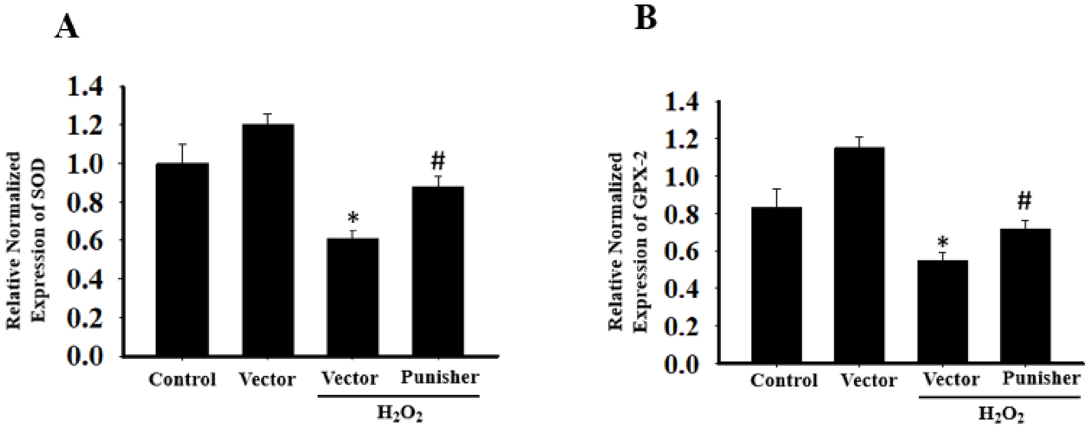

Supplementary Fig. 4. Punisher can regulate antioxidant enzymes. (A) Punisher and netative vector were transfected in VSMC, and mRNA level of SOD was detected by qRT-PCR after 100 mM H_2O_2 stimulation with 24 hours. (B) GPX2 expression was detected via qRT-PCR after 100 mM H_2O_2 stimulation with 24 hours. All values are the average of at least 3 biological replicates, and data shown are the mean±SD. #, is compared with the vector treated with H_2O_2 group, #P < 0.05; *, is compared with the vector group, *P < 0.05.

Figure S5

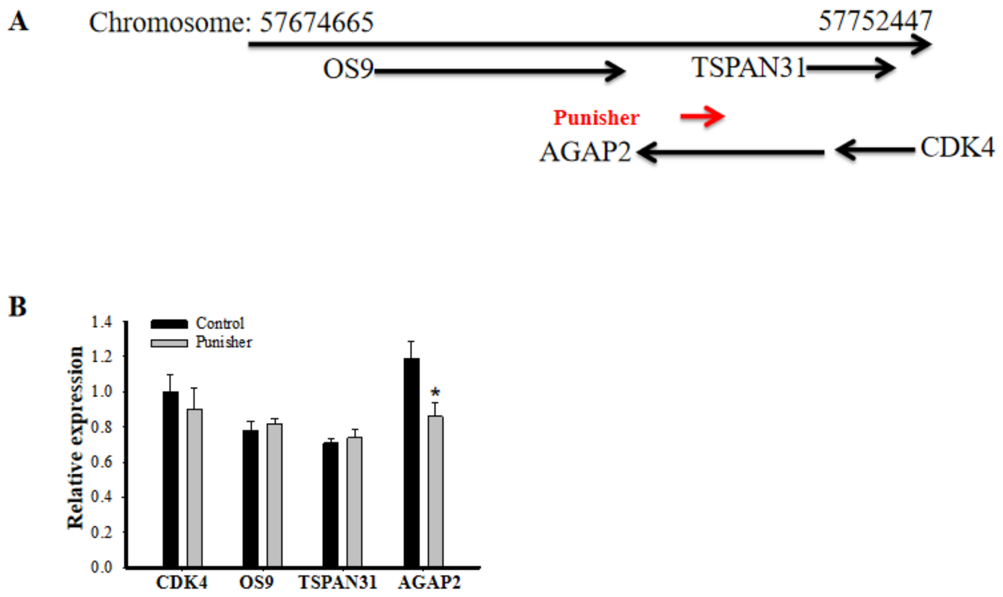

Figure S5. Punisher expression has no relationship with its nearby coding genes. (A) Punisher location in chromosome and (B) expression level of nearby genes after transfection of si-Punisher in VSMCs. Data are shown as mean±s.e.m. of three independent experiments. *$P < 0.05$.

Figure S6

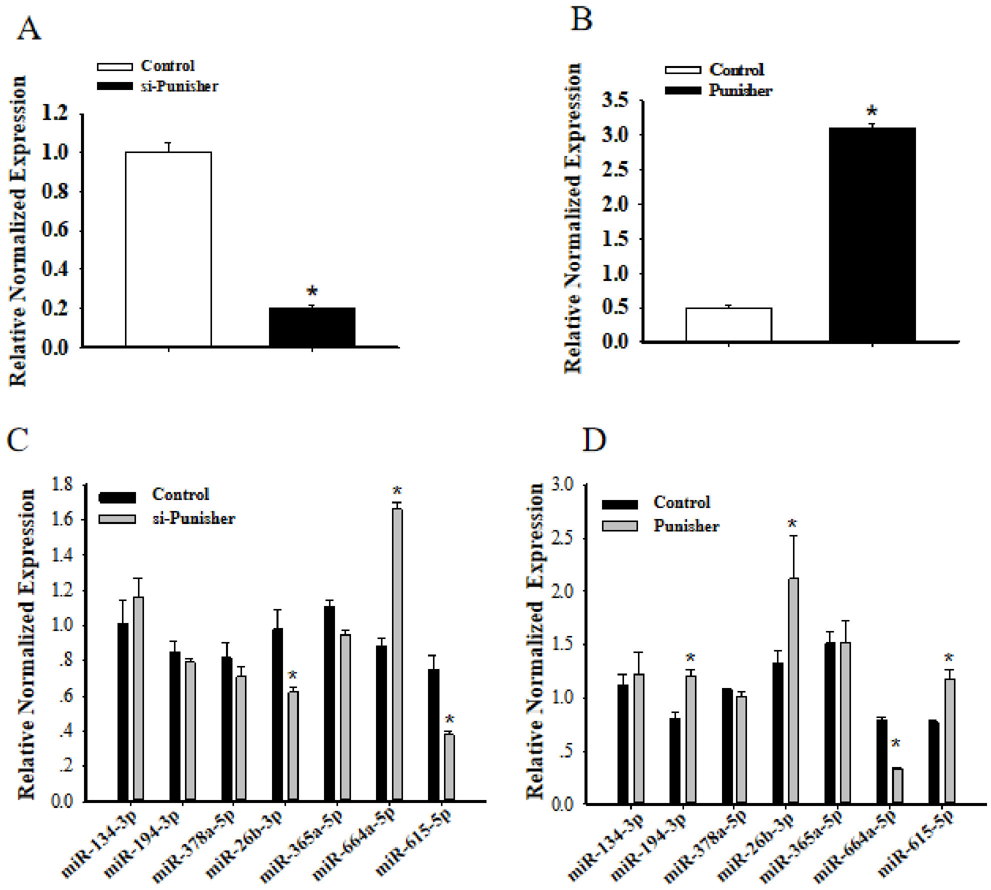

Figure S6. The regulatory effect of Punisher to predicted downstream miRNAs. (A, B) The validation of transfection effeciency of siRNA and overexpression of Punisher in VSMCs. (C, D) The regulatory effect of Punisher to predicted miRNAs. Data are shown as mean ±s.e.m. of three independent experiments. *P < 0.05.

SUPPLEMENTAL TABLES

Table S1. Baseline characteristics of the CAD patients receiving CABG surgery and healthy subjects.

Clinical parameters	Healthy (n = 9)	Disease (n = 12)
Age (yr)	56.14±8.139	61.73±6.018
Men	9(59.3)	7(54.8)
BMI (kg/m2)	22.561±5.1783	23.323±2.8496
Systolic BP (mmHg)	122.56±17.134	141.92±24.267
Diastolic BP (mmHg)	73.24±11.192	85.54±15.961
Total cholesterol(mmol/L)	3.65 ±1.11	4.6315 ±1.291
HDL cholesterol (mmol/L)	1.36±0.52	1.2196±0.337
LDL cholesterol (mmol/L)	1.7429±1.1013	2.8462±1.0159
Triglycerides (mmol/L)	1.20±0.652	1.6835±0.9202
FBG (mmol/L)	4.14±1.5623	5.5180±1.8774
Smoking(%)	4(12.5)	8(45.1)
Diabetes mellitus (%)	0(0)	5(22.6)
Hypertension (%)	0(0)	9(51.6)

Table S2. PCR primers used in this study

Primer name	Sequence (5' to 3')
Punisher-F	CCGGAATTCAGCGCCTAAGGAGC
Punisher-R	CCGCTCGAGTTAAAATCCTTTGGT
has-miR-664a-5p-F	ACTGGCTAGGGAAAATGATTGG
has-miR-378a-5p-F	CTCCTGACTCCAGGTCCTGT
has-miR-365a-5p-F	AGGGACTTTTGGGGGCAGAT
has-miR-194-3p-F	CCAGTGGGGCTGCTGTTATCTG
has-miR-26b-3p-F	CCTGTTCTCCATTACTTGGCTC
has-miR-615-5p-F	GTCCCCGGTGCTCGGATC
has-miR-134-3p-F	CCTGTGGGCCACCTAGTCA
OPA1-F	CCACAGAGAAAGTTAGAGAAATTC
OPA1-R	TTGATAGACTATAGGCAAGAAGAA
AGAP2-F	CAGCCTTTTTGCGAATCGTCG
AGAP2-R	TGCCGTGGGTACTGTGGAT
OS9-F	CTGTCCAGTTTGTTAGGACTGC
OS9-R	GATCCCATAACGCATCTCACTC
TSPAN31-F	CTGCTCCAAGAATGCGCTTTG
TSPAN31-R	CAATGACTCCGCCGATGATGT
CDK4-F	AGCTGGTCACATGGTGAGG
CDK4-R	CCATAGGCACCGACACCAAT
FIS1-F	CAAGGAACTGGAGCGGCTCATT
FIS1-R	GGACACAGCAAGTCCGATGAGT
SOD2-F	CCCAGATAGCTCTTCAGCCTGCACT
SOD2-R	TAAGCGTGCTCCCACACATCAATCC
GPX2-F	ATTTGGACATCAGGAGAACTGT
GPX2-R	CTTCAGGTAGGCGAAGACA
GAPDH-F	GTCTCCTCTGACTTCAACAGCG
GAPDH-R	ACCACCCTGTTGCTGTAGCCAA
β-Actin-F	CACCATTGGCAATGAGCGGTTC
β-Actin-R	AGGTCTTTGCGGATGTCCACGT
U6-F	CTCGCTTCGGCAGCACA

U6-R	AACGCTTCACGAATTTGCGT
